# The Influence of the Structural Architecture on the Swelling Kinetics and the Network Behavior of Sodium-Alginate-Based Hydrogels Cross-Linked with Ionizing Radiation

**DOI:** 10.3390/gels10090588

**Published:** 2024-09-12

**Authors:** Ion Călina, Maria Demeter, Gabriela Crăciun, Anca Scărișoreanu, Elena Mănăilă

**Affiliations:** Electron Accelerators Laboratory, National Institute for Laser, Plasma and Radiation Physics, 409 Atomiștilor St., 077125 Măgurele, Romania; calina.cosmin@inflpr.ro (I.C.); maria.demeter@inflpr.ro (M.D.); gabriela.craciun@inflpr.ro (G.C.)

**Keywords:** superabsorbent hydrogel, structural architecture, high swelling degree, water diffusion, e-beam radiation cross-linking

## Abstract

The present work discusses the influence of the structural architecture of sodium alginate–co-acrylic acid–poly(ethylene) oxide hydrogels, crosslinked through electron beam (e-beam) radiation processing. The most important properties of the hydrogels were studied in detail to identify a correlation between the architecture of the hydrogels and their properties. Furthermore, the effect of sodium alginate (NaAlg) concentration, the amounts of the polymer blend, and the size of the samples on hydrogel properties were investigated. The results show that the hydrogels cross-linked (0.5% and 1% NaAlg) with 12.5 kGy exhibit improved physicochemical properties. High gel fraction levels (exceeding 83.5–93.7%) were achieved. Smaller hydrogel diameter (7 mm) contributed to a maximum swelling rate and degree of 20.440%. The hydrogel network was dependent on the hydrogels’ diameter and the amount of polymer blend used. The hydrogels best suited the first-order rate constants and exhibited a non-Fickian diffusion character with diffusion exponent values greater than 0.5. This study indicates that the cross-linked hydrogel has good properties, particularly because of its high degree of swelling and extensive stability (more than 180 h) in water. These findings show that hydrogels can be effectively applied to the purification of water contaminated with metals, dyes, or even pharmaceuticals, as well as materials with a gradual release of bioactive chemicals and water retention.

## 1. Introduction

Superabsorbent hydrogels are novel functional polymer materials with hydrophilic functionalities and a three-dimensional network structure. Due to their nature, they have a unique water absorption and retention capability [1]. As a result, they are widely used in various applications, including biomedicine, drug release, agriculture, horticulture, healthcare, and everyday life supplies [2]. Chemically cross-linked hydrogels are more stable and have a longer degradation time than physically cross-linked hydrogels. The presence of physical bonds in the polymer matrix is specific to polymer materials with reversible properties but with a short lifetime and poor mechanical properties. Although initiators are required for the chemical cross-linking of hydrogels or other commercial products, e-beam irradiation does not require the addition of any initiators to the reaction mixture, thus preventing the formation of unwanted products. Environmental protection, cost-effective processing, and product quality are all improved by the creation of chemical bonds through grafting, polymerization, and cross-linking reactions generated by ionizing radiation.

When using irradiation technology, water undergoes radiolysis, which causes chemical reactions to start in aqueous media and produce a number of reactive species. Molecular products and radical species with different reactivities, such as e^−^, H⋅, HO⋅, H_2_O_2_, H_2_, and H_3_O^+^, are produced. Depending on the concentration of the polymer, the H⋅ and HO⋅ radicals interact rapidly with polymeric chains through hydrogen abstraction, resulting in the creation of a series of macroradicals [3]. A greater number of free radical active sites will arise on the biopolymer chain when the irradiation dose is increased, which will cause the most monomers/polymers to establish permanent bonds on the biopolymer chain [4,5].

Thus, the synthesis of hydrogels using e-beam radiation crosslinking leads to the creation of a homogenous network structure [6].

Radiation processing is a suitable alternative, providing simultaneous cross-linking and sterilization in a single step and allowing for control over the physicochemical properties of hydrogels by combining the benefits of ionizing radiation along with the polymer properties. The swelling behavior, the mechanical properties of the hydrogel, and the process of water transport across the hydrogel network are all significantly impacted by the cross-linking density of polymer hydrogels, which can be tailored by adjusting the irradiation dose. Furthermore, when suitable experimental irradiation conditions and doses are employed, it has been demonstrated that irradiation does not influence the biocompatibility of polymers [7]. To highlight the degree of crosslinking produced by irradiation, it is essential first to investigate the yield of the crosslinking reaction, as well as the swelling characteristics of the newly obtained hydrogel.

The e-beam cross-linking method is superior for creating sodium alginate-co-acrylic acid-poly(ethylene) oxide hydrogels. Its ability to produce good-quality hydrogels under controlled conditions and the absence of residual chemicals makes it an excellent choice over chemical and UV-based crosslinking techniques. The e-beam cross-linking often produces an increased and homogeneous gel fraction. E-beam irradiation generates free radicals equally over the hydrogel matrix, resulting in more widespread and consistent crosslinking. This consistency ensures that a greater proportion of the hydrogel remains insoluble, resulting in a higher gel fraction and good swelling behavior. Chemical cross-linking initiators often produce less-uniform gel fractions due to the uneven distribution of crosslinking agents and incomplete reactions, which can result in lower overall gel fractions and heterogeneous swelling [8]. UV-crosslinking mediated by photoinitiators may also produce lower gel fractions—especially in thicker samples—due to limited light penetration, leading to non-uniform crosslinking and heterogenous swelling degrees.

Monitoring the change in dry hydrogel mass or polymer volume as a function of time in the presence of an appropriate solvent yields swelling curves [9]. The size of the hydrogel strongly affects its properties and characteristics, including its ability to swell, its ability to transport water, its mechanical strength, and the rate and amount of drugs or nutrients that are loaded and released into and out of their network. A hydrogel’s size can be readily adjusted to produce materials with exact specifications that are appropriate for a variety of biological, medicinal, environmental [10], and agricultural uses [11,12].

The potential of NaAlg to form hydrogels is essential for a variety of uses in the pharmaceutical [13], regenerative medicine [14], and food industries [15]. Usually, when monovalent cations interact with free carboxylic acid groups of NaAlg, they form weak interactions that result in the formation of soluble salts. Conversely, divalent and multivalent cations typically generate strong ionic interactions, which result in a strong and highly stable gelation [16].

An additional benefit of the NaAlg hydrogels is their ease of manufacturing, which is beneficial from an industrial and scientific viewpoint. Hydrogels based on poly(ethylene oxide) (PEO) are thought to be biocompatible, have minimal toxicity, and are elastic and flexible because of their hydrophilic nature, which closely resembles the behavior of natural compounds [17]. PEO has attracted the interest of researchers mainly because of its distinct characteristics in solution; it has uses in many different sectors, such as wound therapy [18], drug delivery [19] and tissue engineering [20]. 

Hydrogels can be engineered via various techniques, and their applicability and end-use are determined by their qualities, size, architecture, structure, and type of cross-link. Recent research indicates that the hydrogel’s sizes and geometries are crucial to its application because single-structure hydrogels are unable to fulfill all demands. In a recent review work, Peters et al. described the primary hydrogel synthesis techniques that establish the architecture and structural properties of these materials. After reviewing the literature, they concluded that the preparation techniques and real application in interaction with natural or biological fluids are the most crucial elements of hydrogel architecture [21]. However, there are a lot of significant aspects of hydrogel architecture that need to be addressed. 

To our knowledge, no one has investigated the impact of irradiation-induced hydrogel architecture on swelling kinetics. Furthermore, without considering their dimensions, it is impossible to define the unique swelling properties of hydrogels exactly. Only the architecture of the polymer network (molecular and supramolecular) and its influence on the swelling behavior of the hydrogels were studied. The molecular level includes the structure of the monomer: the functional groups that are involved in the polymerization and crosslinking reactions; the functional groups that influence the swelling behavior (amino, carboxyl, hydroxyl, etc.). The supramolecular level involves a 3D structure of monomers and chain segments that results in measurable properties such as mesh size, crosslink density, and porosity [22]. 

The current work contributes to the development of hydrogels with increased capacity to maintain humidity and developing hydrogels for the delivery and storage of nutrients. Additionally, the current work is a methodical investigation that advances the state of the art in the field of hydrogel swelling qualities. The effects of the hydrogel architecture, diameter, and composition on the absorption capacities and kinetics were also investigated. In addition to this primary goal, the paper focuses on establishing a relationship between the preparation technique and the architecture of the newly developed hydrogels, as well as how the hydrogel size affects their characteristics and the kinetics of water transport inside them. The physicochemical characteristics of the hydrogel, including gel fraction, swelling, water diffusion, porosity, and network parameters were examined to clarify these goals and assess the hydrogel’s suitability for different uses.

## 2. Results and Discussion

### 2.1. Hydrogels Formation

**Figure 1:**
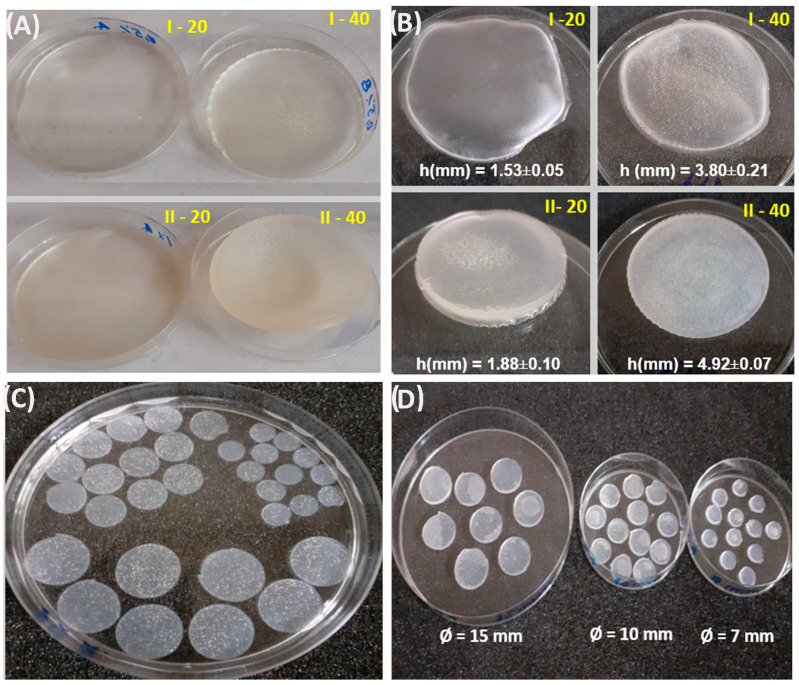
Representative images of hydrogels: (**A**) after e-beam irradiation at room temperature (25 °C)*;* (**B**); after being stored for 24 h at ambient temperature; (**C**) cut and immersed in ethanol for 24 h; (**D**) cut into discs at 15, 10, and 7 mm in diameter.

The hydrogels were produced using e-beam radio-induced cross-linking at a dose of 12.5 kGy. The composition of the hydrogels varied through the use of different concentrations of NaAlg according to Table 6. To highlight the effect of irradiation on the polymer composition, different volumes of polymer mixture were used. Also, to highlight the impact of the size of the hydrogels on the swelling capacity, the kinetics of water transport, and on the structural parameters, the cross-linked hydrogels were cut into discs with different diameters (Figure 1). Additionally, the cross-linked hydrogels were sliced into discs with varying diameters to emphasize the effect of hydrogel size on swelling capacity, water transport kinetics, and network characteristics (Table 1).

### 2.2. Gel Fraction

**Figure 2:**
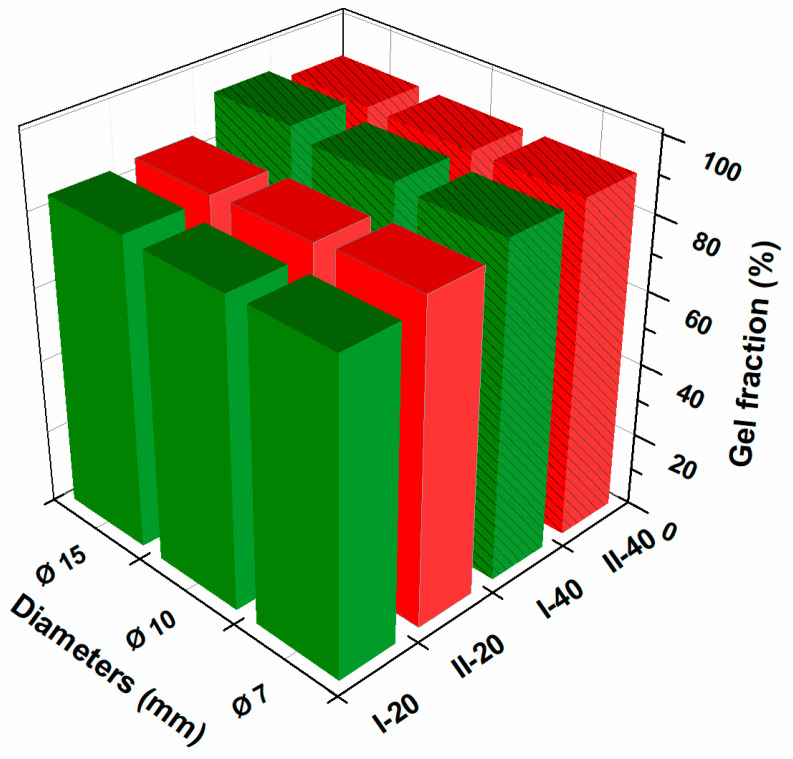
The effect of NaAlg concentration, polymer volume, and hydrogel size on the gel fraction.

The effects of the diameter, the volume of polymeric solution, and the concentration of NaAlg on the gel fraction of hydrogels are shown in Figure 2 and Table 2. For cross-linked hydrogels, lower gel fractions (83–88%) were achieved at a volume of 20 mL of polymer mixture. Gel fractions over 90% were obtained with larger quantities of polymer mixture. The data obtained indicate that there are also significant variations in the gel fraction values due to the change in the NaAlg concentration from 0.5% to 1%. The hydrogel diameter does not appear to have a major effect on the gel content.

It is important to note that increased gel fraction values result from effective e-beam crosslinking of the NaAlg, AA, and PEO polymers in the presence of the K_2_S_2_O_8_ initiator. Macromolecular radicals are produced directly or indirectly from the polymer chain through the e-beam crosslinking process by reactive species (HO· and H· radiation-induced radicals) in the surrounding environment. Similar to our obtained results, Mohamady Ghobashy et al. prepared NaAlg-polyacrylamide hydrogels via γ-radiation at 20 kGy and demonstrated that the gel content decreased from 87% to 66% with increasing NaAlg content from 0.5 to 1.0% [23]. Meanwhile, Bardajee et al. synthesized superabsorbent hydrogels based on PDMAEMA-NaAlg, also through the use of γ-radiation at doses of 12 kGy. They obtained lower gel fraction values (48%, 78%) at 0.5% and 1% NaAlg concentrations, respectively [24].

### 2.3. Network Parameters

**Figure 3:**
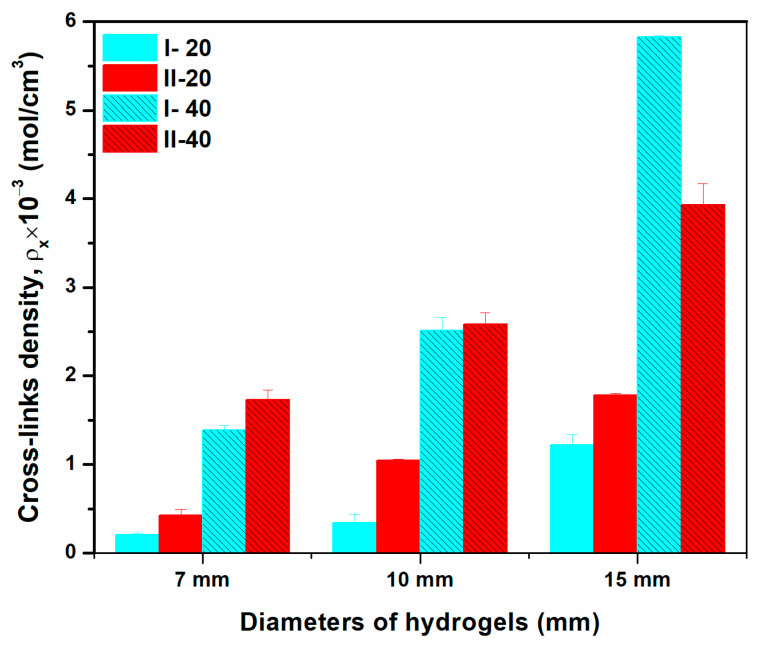
The cross-links density of the hydrogel samples.

Determining the network characteristics and monitoring macromolecule or nutrient movement via the polymer network is essential [25]. The network parameters $\nu_{2s}$, $M_{c}$, $\rho_{x}$, and ξ and their numerical values are shown in Table 2. In general, mesh size increases as hydrogel diameter decreases and is two times smaller for crosslinked samples prepared with 40 mL of polymer mixture. Stronger cross-links are formed when ξ values decrease as solution concentration rises. As the volume of polymer solution increased from 20 to 40 mL, the Mc for hydrogel composition I (0.5% NaAlg) collapsed from 60.79–358.2 kg/mol to 12.7–53.07 kg/mol, resulting in a more compact polymer network. When more polymer solution was used, more radicals were generated; consequently, Mc lowered and $\rho_{x}$ values increased (Figure 3). The gel fraction and swelling behavior also confirm the results since increasing cross-link density causes an increase in gel fraction and a decrease in swelling degree. The numerical data regarding the porosity of the hydrogels under study is displayed in Table 2. The porosity indicates the absorption performance of polymer-based hydrogels, and generally, a high porosity promotes absorbent transport and offers a large specific surface area. There was no apparent distinction in porosity between the hydrogels generated with 40 mL of solution and those with 20 mL; the hydrogels with 40 mL of solution had the lowest porosity. The cross-linking density data indicate that the hydrogels’ increased porosity, which ranged from 97.83% to 99.51%, is the result of cross-linked structures. Porous channels are created and the porous structure is stabilized by the hydrogen bonding and interconnectivity in cross-linked hydrogels. 

Results shows that increasing thickness give a significant increase in gel fraction and cross-linking degree for all discs. Also, there is a large reduction in mesh size and swelling with increasing thickness of sample. The swelling observation indicates that inter plenary network (IPN) formation of high thickness hydrogel (I-40 and II-40) is denser than the small thickness hydrogel (I-20 and II-20). 

Also, in this case, whereby water is the main constituent of hydrogel (>78%), it is believed that there is a reduction in the OH functional group capability to attract water molecules. Similar results were obtained in other studies on gel samples based on sago starch [26,27]. 

### 2.4. Swelling Behavior

**Figure 4:**
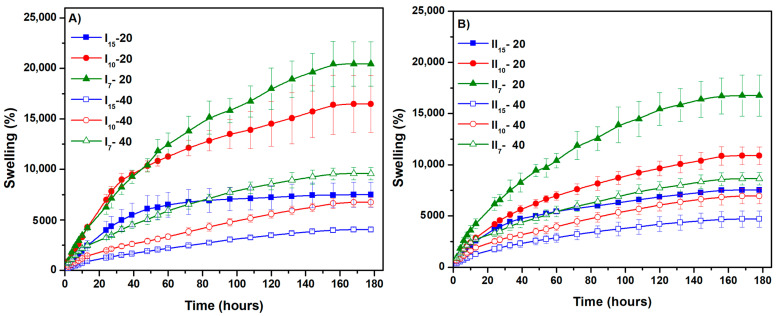
Relationship between the amount of polymer solution and swelling degree of hydrogels at different sizes: (**A**) for hydrogels with a NaAlg concentration of 0.5% and (**B**) for hydrogels with a NaAlg concentration of 1%.

Figure 4A,B show the time variation of the degree of swelling at the equilibrium of the NaAlg hydrogels. A concentration of 0.5% NaAlg leads to hydrogels with a maximum swelling degree of about 20,000%. At 1% NaAlg, the degree of swelling at equilibrium is slightly lower; however, all experimental data demonstrate that the superabsorbent character of hydrogels based on NaAlg is maintained. The highest degree of swelling is obtained for the hydrogel sample with the smallest diameter taken for analysis. 

Unexpectedly, at larger amounts of pre-hydrogel subjected to e-beam irradiation under the same conditions, from the point of view of the swelling properties, a more pronounced crosslinking effect is evident. At the same time, the diameter of the hydrogel sample (obtained after e-beam irradiation) significantly influences the swelling. 

**Figure 5:**
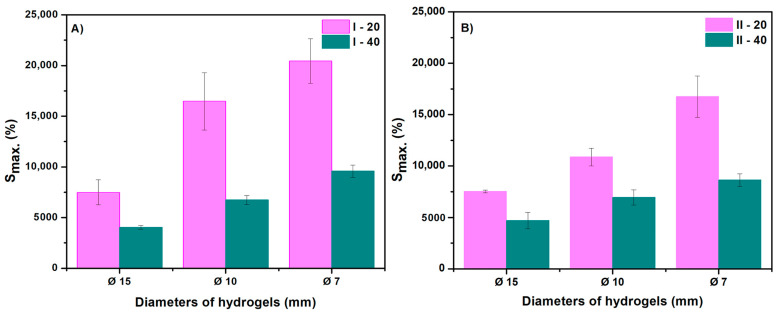
The swelling degree values of the hydrogels based on size: (**A**) for hydrogels with a NaAlg concentration of 0.5% and (**B**) for hydrogels with a NaAlg concentration of 1%.

**Figure 6:**
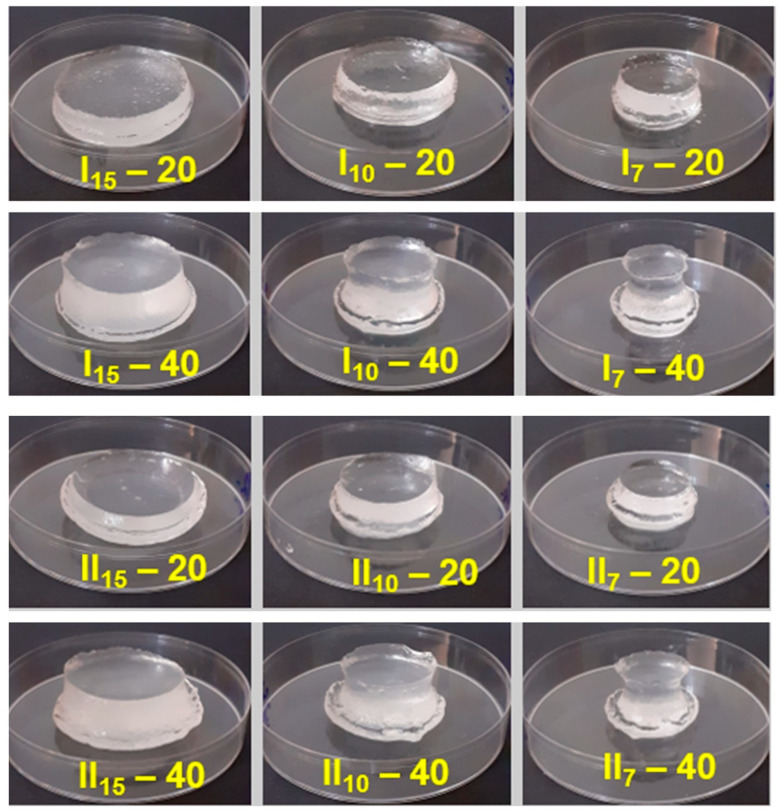
The hydrogel samples swelled at equilibrium.

In all cases (Figure 5A,B), it was observed that with the increase in the diameter of the sample, swelling decreased significantly. For example, at a diameter of 15 mm, the swelling decreases to approximately 5000%, and at a diameter of 7 mm, the hydrogel exhibits swelling of approximately 20,000% (Table 3). At the same time, the hydrogels maintain their structure and absorb liquid for 180 h (Figure 6).

The newly developed hydrogels show an equilibrium water content (EWC (%)) of over 98%, which suggests that the combination of hydrophilic polymers and their concentrations in the polymer mixture were well chosen, as well as the radiation doses, leading to obtaining a hydrogel with superabsorbent properties (Table 3).

### 2.5. Swelling Kinetics Study

**Figure 7:**
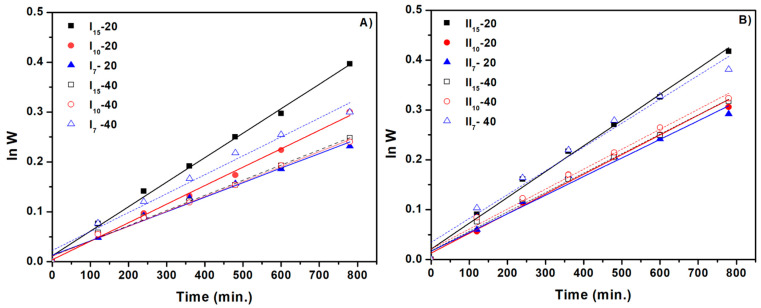
The first-order swelling kinetics of cross-linked hydrogels: (**A**) for hydrogels with a NaAlg concentration of 0.5% and (**B**) for hydrogels with a NaAlg concentration of 1%.

Equation (12) was used to plot lnW versus t in Figure 7, and the slope of the plotted straight line was used to compute k_1,s_. The findings are displayed in Table 4. Hydrogel samples with a large diameter (10–15 mm) and a 40 mL solution volume have been found to have a lower water absorption rate constant than hydrogel samples prepared with a 20 mL volume.

**Figure 8:**
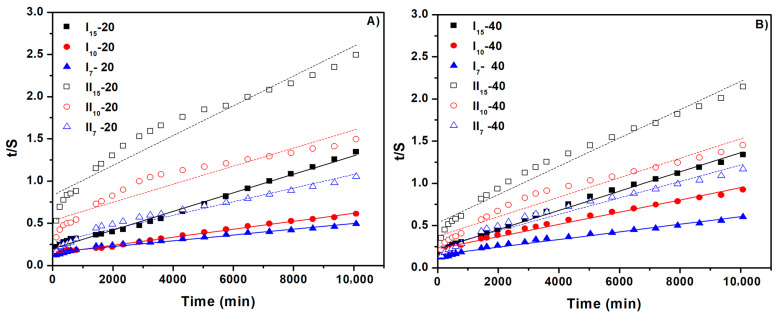
The second-order swelling kinetics of cross-linked hydrogels: (**A**) for hydrogels with a NaAlg concentration of 0.5% and (**B**) for hydrogels with a NaAlg concentration of 1%.

According to these findings, which are in line with the swelling degree data, the water absorption rate constant in the 20 mL hydrogel samples is larger than that of the 40 mL hydrogel samples. Figure 8 shows the straight line obtained from plotting t/S versus t. The slope and intercept of the plotted consecutive lines were used to determine Seq and k_2,s,_ respectively, and the values of these parameters are shown in Table 4. The highest Seq values were achieved at the smallest diameter of 7 mm. The theoretical equilibrium swelling capacities (Seq) were similar to the experimental values. Additionally, when the hydrogel’s diameter increased, the swelling rate constant (k_2,s_) dropped. This is owing to the interconnected three-dimensional structure of the hydrogels, which slows the diffusion of aqueous solutions through the hydrogel network, extending the time to reach equilibrium swelling capacity and consequently reducing the swelling rate.

The water transport mechanism in hydrogels is evaluated according to parameter n. For n = 0.5, the hydrogel shows a Fickian water transport mechanism characterized by a controlled diffusion. If, 0.5 < n < 1, the so-called anomalous diffusion transport mechanism (non-Fickian) occurs in the hydrogel. If n = 1, the hydrogel shows Case II diffusion, and when n >1, it shows super Case II diffusion. The parameter n is determined using the first 60% of the experimental swelling data. 

**Figure 9:**
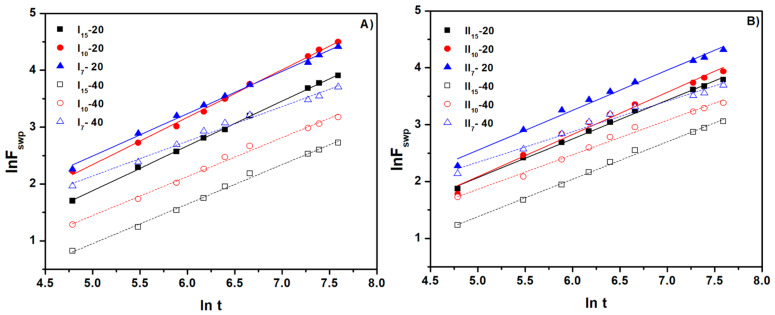
The swelling kinetic curve of the cross-linked hydrogels (ln F vs. ln t): (**A**) for hydrogels with a NaAlg concentration of 0.5% and (**B**) for hydrogels with a NaAlg concentration of 1%.

**Figure 10:**
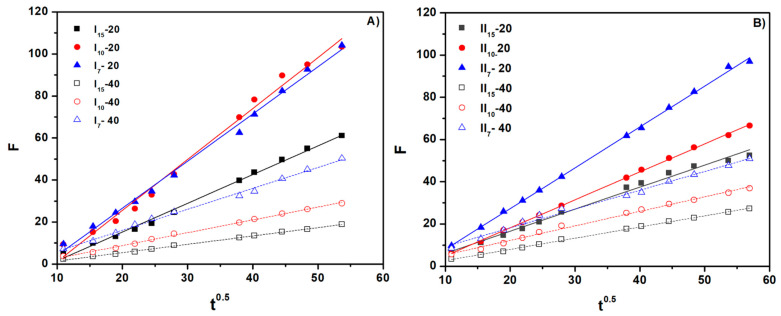
The swelling kinetic curve of the cross-linked hydrogels (F vs. t^0.5^): (**A**) for hydrogels with a NaAlg concentration of 0.5% and (**B**) for hydrogels with a NaAlg concentration of 1%.

All hydrogels present n > 0.5, showing an anomalous transport mechanism Case II diffusion. Plots of ln F vs. ln t and F vs. t^0.5^ were obtained from experimental data processing through linear fitting. The parameters n and D are determined from the slope of the graph lines. From Figure 9 and Figure 10, as well as from Table 5, it can be seen that the values of the n and D parameters obtained by linear fitting have an increased correlation coefficient of over 0.99 [28].

As is shown in Table 5, the water transport mechanism (n) in hydrogels is governed by an anomalous diffusion transport. A non-Fickian mechanism demonstrates that the relative speed of diffusion and the relaxation of the macromolecular chains control the transport of the fluid inside the hydrogel network. These findings suggest that the diffusion of water onto the hydrogels is hindered by relatively high crosslinking density levels. The values for n demonstrate that non-Fickian transport of water into the hydrogels is evident and that swelling decreased with increasing crosslink density. This is generally explained as a consequence of the slow relaxation rate of the polymer. The reason for this is probably attributable to the intramolecular hydrogen bonding between carboxylic acid of poly(acrylic acid); thus. the numbers of hydrophilic groups of the gels decrease. The presence of hydrogen bonding in the copolymer matrix caused the network to be less swollen [29]. 

Diffusion coefficients (D) of NaAlg hydrogels varied from 0.931 to 2.787 cm·s^−1^ at 0.5–1% NaAlg. Hydrogels with higher diameters (15 mm) showed increased diffusion coefficients. Table 5 displays data indicating that the hydrogels containing 20 mL polymer solution had higher diffusion coefficients than those containing 40 mL polymer solution.

This indicates that the 20 mL hydrogel network exhibits faster diffusion of water molecules compared to the 40 mL hydrogels. Moreover, the hydrogels produced from 40 mL of polymer solution show a significant decrease in their diffusion coefficients, indicating that they do not absorb additional water, a feature supported by the swelling data. 

### 2.6. Chemical Structure Analysis (FTIR)

**Figure 11:**
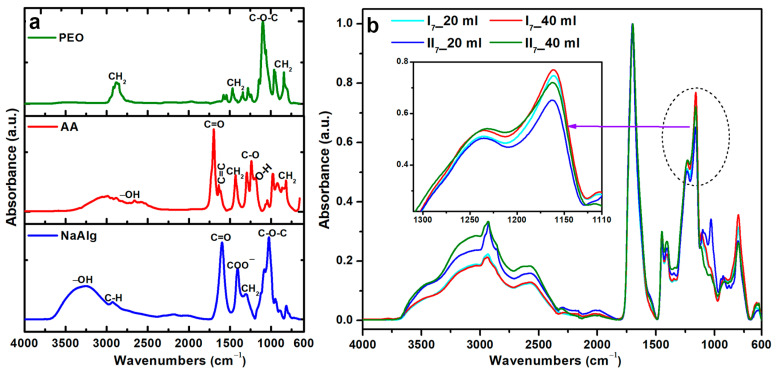
FTIR spectra of (**a**) native polymers (NaAlg, AA/ and PEO) and (**b**) I (0.5% NaAlg) and II (1% NaAlg) hydrogels with a diameter of 7 mm.

The FTIR spectra of native polymers (NaAlg, PEO and AA) are presented in Figure 11a. The NaAlg spectrum showed the principal bands at 3260 cm^−1^ (νO–H), 2927 cm^−1^ (νC–H), 1598 cm^−1^ (νC=O), 1408 cm^−1^ (δCOO^−^), 1305 cm^−1^ (δCH_2_), 1123 cm^−1^ (νC–O–C), 1026 cm^−1^ (νC–O–C), and 947 cm^−1^ (νC–O) [29]. For AA, the major peaks were identified at 2888/2660/2583 cm^−1^ (νO–H), 1697 cm^−1^ (νC=O), 1635/1615 cm^−1^ (νC=C), 1432 cm^−1^ (δCH_2_), 1238 cm^−1^ (νC–O), and 1184 cm^−1^ (νO–H) and 978 cm^−1^ (δCH_2_) [30]. The FTIR analysis of PEO showed: 2916/2850 cm^−1^ (νCH_2_), 1466/1340 cm^−1^ (δCH_2_), 1097 cm^−1^ (νC–O–C), and 960/840 cm^−1^ (δCH_2_) [31].

The characteristic peaks of the cross-linked hydrogels were as follows: 2933 cm^−1^ (νC–H), 2590 cm^−1^ (νO–H), 1701 cm^−1^ (νC=O), 1450 cm^−1^ (δCH_2_), 1408 cm^−1^ (δCOO^−^), 1230 cm^−1^ (νC–O), 1164 cm^−1^, ^1^ (νO–H), 1095 cm^−1^ (νC–O–C), 1032 cm^−1^ (νC–O–C), 931 cm^−1^ (δCH_2_).

The spectra shown in Figure 11b highlight the successful cross-linking of the hydrogels. The hydrogels containing 1% NaAlg displayed a shift in their FTIR spectra to higher wavenumbers, ranging from 2933 cm^−1^ for 20 mL to 2993 cm^−1^ for 40 mL of polymeric solution. Furthermore, it was noted that the bands at 2603 cm^−1^, 1408 cm^−1^, and 1230 cm^−1^ had increased in intensity.

The cross-linking reaction of NaAlg, PEO, and AA polymers corresponds to the shift to higher wavenumbers of the FTIR peaks in the 3000–2550 cm^−1^ range. Moreover, a reduction in band intensity in the 1300–1100 cm^−1^ region emphasizes the substantial crosslinking of the hydrogel achieved with 1% NaAlg at 40 mL of polymeric solution. This observation is also supported by the increase in cross-linking density and the decrease of 20% of the swelling capacity. The amount of the polymer mixture and the NaAlg content have an impact on the hydrogel final FTIR bands intensity. Also, these parameters directly influence the structure of the hydrogels, as demonstrated by the swelling kinetics analysis and the mesh size experimental results. The hydrogel structural stability is further demonstrated by the presence of the polymer-specific peaks following e-beam cross-linking. 

### 2.7. Morphology of Hydrogels

**Figure 12:**
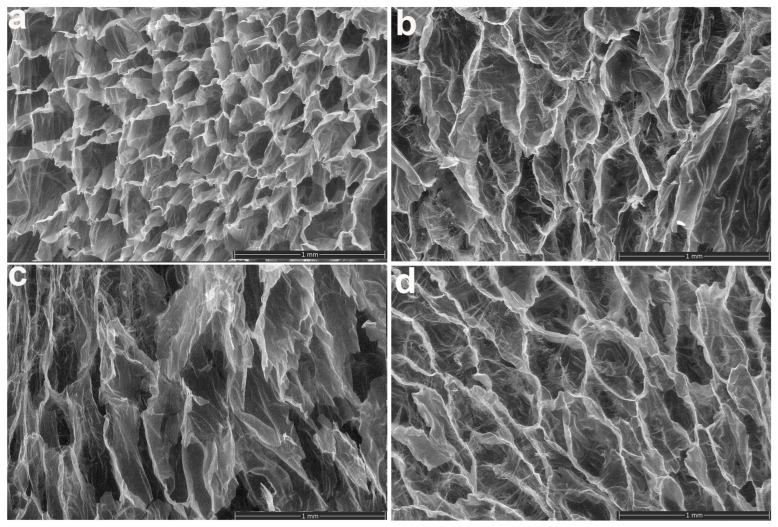
SEM images of the (**a**) I_7__20 mL, (**b**) I_7__40 mL, (**c**) II_7__20 mL, and (**d**) II_7__40 mL hydrogels at 50× magnification. Scale bars indicate 1 mm.

Figure 12 shows the representative SEM images of hydrogels prepared with different amounts of polymer solution (20 mL and 40 mL) and different concentrations of NaAlg (0.5–1%) at a dose of 12 kGy. By adjusting the polymers concentration, hydrogels with various pore sizes and implicitly with different levels of crosslinking can be produced. The size of the hydrogel pores and their even distribution across the hydrogel structure determine not only the architectural changes that take place during the swelling process but also the fluid transport within these networks.

At 0.5% NaAlg, hydrogels with macroporous structures, interconnected pores, and homogenous structures are obtained. The SEM measurements highlight the fact that at 1% NaAlg and 40 mL of polymeric solution, hydrogels with a dense and compact structure are obtained, in which the existence of a small number of pores is observed, with walls with thickened outer walls. The increased crosslinking density allows hydrogels to maintain their porous structure but creates irregularly shaped and heterogeneous structures. 

These results exhibit a strong correlation with the swelling experiments and the network parameters, which demonstrate that an increase in polymer content also leads to a decrease in swelling degree and pore size reduction.

## 3. Conclusions

This work developed sodium alginate–acrylic acid–polyethene oxide hydrogels crosslinked by e-beam irradiation at a dose of 12.5 kGy. The study demonstrated that the physicochemical characteristics and swelling kinetics of the hydrogels were impacted by their size and content. The crosslinked hydrogels have demonstrated remarkable water absorption efficiency. 

The results have shown that changes in NaAlg content and hydrogel size can cause swelling from 4440 to 20.440%. The composition of the hydrogel and the degree of cross-linking, which were impacted by the hydrogel samples’ diameter and thickness, governed the swelling behavior. Additionally, the gel content rises with the amount of polymer mixture, but it remains constant for 40 mL of polymer solution at a concentration of 1% NaAlg. As the hydrogel reached the equilibrium swelling degree, it was observed that the pore size varied between 26–266 nm. 

The SEM analysis of hydrogels reveals a strong relationship between polymer concentration, crosslinking density, and the resulting hydrogel structure. This highlights the critical role of polymer concentration and solution volume in adjusting the hydrogel properties, particularly for applications where controlled swelling and specific pore structures are required. Therefore, the pore size decides the diffusion rate throughout the three-dimensional porous structure of the hydrogel and the time required for the hydrogel to reach equilibrium regardless of the fluid. The swelling degree was not uniform, so it was demonstrated that structures with larger volumes of polymer may have different pore sizes than structures with smaller volumes, or the structure may take longer to reach equilibrium. The cross-linked hydrogels have n values ranging from 0.53 to 0.83, indicating non-Fickian diffusion as the principal mechanism of water transport. 

The FTIR analysis of the hydrogels confirms successful cross-linking between NaAlg, PEO, and AA polymers. The results demonstrate that higher NaAlg content and higher volumes of polymer solution lead to denser crosslink networks, which are more structurally stable, as evidenced by FTIR spectra after e-beam crosslinking.

In conclusion, taking into account the fact that the degree of swelling increases as the hydrogel’s diameter decreases, we can state that our findings imply that these hydrogels are excellent for a variety of applications since they provide an appropriate degree of swelling and indicate low polymer concentrations.

## 4. Materials and Method

### 4.1. Materials

Sodium alginate (NaAlg, Mw = 120,000–190,000 g/mol), viscosity = 15–25 cP, (1% in water), acrylic acid (AAc, Mw = 71.08 g/mol, density = 1.13 g/cm^3^), poly(ethylene oxide) (PEO, Mw = 300,000 g/mol, density = 1.210 g/cm^3^), and potassium persulfate (K_2_S_2_O_8_, Mw = 270.322 g/mol, density = 2.477 g/cm^3^) were purchased from Merck KGaA, Darmstadt, Germany.

### 4.2. Sample Preparation

Two solutions of (0.5–1%) NaAlg were dissolved in 100 mL double-distilled water via mechanical stirring at room temperature. Following homogeneity, incorporate and add 20% AAc and 0.1% PEO. After the polymers were completely dissolved, 0.1% of K2S2O8 was added. The detailed compositions are presented in Table 6.

### 4.3. E-Beam Cross-Linking

Different volumes of polymer solution (20 mL and 40 mL, respectively) were poured into Petri dishes (Ø = 90 mm), as detailed in Table 7. The e-beam cross-linking was performed at room temperature (25 °C) with a dose of 12.5 kGy and a dose rate of ~1 kGy/min. After e-beam irradiation, the Petri dishes were left at room temperature for 24 h (Figure 11a,b). The hydrogels were cut into discs (15, 10, and 7 mm in diameter) using an in-house device, as shown in Figure 1D. The hydrogel discs were initially washed with ethanol to remove unreacted components (Figure 1C) and then dried for 24 h in an oven at 50 °C to constant mass. A 5.5 MeV linear electron accelerator (ALID-7) from the Electron Accelerators Laboratory of the National Institute for Lasers, Plasma, and Radiation Physics (INFLPR), Măgurele, Romania, was used for e-beam cross-linking. The following parameters were established: a beam current of 5 μA, an electron pulse repetition rate of 53 Hz, an irradiation area of 10×10 cm^2^, and a distance between the exit window and sample h = 124 cm. The conical-shaped beam is oriented vertically and reaches perpendicular to the pre-hydrogel polymeric solution poured into the Petri dish. The nominal dose and absorbed dose were measured using calibrated graphite calorimeters and B3 radiochromic films against alanine reference dosimeters.

### 4.4. Characterization

#### 4.4.1. Gel Fraction

After drying in an oven at 50 °C, the weight of cross-linked hydrogels (*w_o_*) was measured. Then, the samples were swelled in double-distilled water at room temperature for 48 h to obtain the insoluble hydrogels, dried at 50 °C, and weighed again (*w_d_*). The percentage of gel fraction (%) was calculated by Equation (1) [32].

(1) $$G\;(\%)=\frac{w_{d}}{w_{i}}\times 100.$$

#### 4.4.2. Determination of Network Structure

The cross-linked network structure of hydrogel samples has been evaluated using Equations (2)–(6), including the cross-link density (*ρ_x_*), the ratio between the molecular weight of the polymer repeating units (*M_r_*), the average molecular weight between cross-links (*M_c_*), the Flory–Huggins interaction parameter (χ), and the mesh size (ξ) [4,33,34,35].

(2) $$\rho_{x}=\frac{M_{c}}{M_{r}},$$

(3) $$M_{r}=\frac{\left(m_{NaAlg}\times M_{NaAlg}\right)+\left(m_{AA}\times M_{AA}\right)+\left(m_{PEO}\times M_{PEO}\right)}{m_{NaAlg}+m_{AA}+m_{PEO}},$$

(4) $$M_{C}=-V_{1}d_{P}\frac{\nu_{S}^{\frac{1}{3}}-\frac{\nu_{S}}{2}}{\ln(1-\nu_{S})+\nu_{S}+\chi \nu_{S}^{2}},$$

(5) $$\chi =0.431-0.311\nu_{S}-0.036\nu_{S}^{2},$$

(6) $$\xi =\upsilon_{s}^{-1/3}\cdot l\sqrt{\frac{2C_{n}M_{c}}{M_{r}}},$$

where *V_s_* is the molar volume of the swelling agent (distilled water, 18 cm^3^/mol); *d_p_* is the density of hydrogel; *υ_s_* (cm^3^) is the polymer volume fraction in the swollen state; *l* is the length of the C–C bond (0.154 nm); *C_n_* is the Flory characteristic ratio (AA = 6.7) [36]; NaAlg = 21.1 [37]; PEO = 4.98 [38]. Using Equation (7), the porosity of the hydrogels was determined as a function of the volume ratio of water at equilibrium (*V_d_*) [33]:

(7) $$P(\%)=\frac{V_{d}}{1-V_{d}}\times 100.$$

#### 4.4.3. Swelling Behavior

The dried hydrogel samples with w_d_ weight were swollen in double-distilled water at room temperature. Swollen hydrogel weight was measured precisely (*w_s_*) at specified time intervals after removing excess water on the hydrogel surface. After 180 h, the swelling degree was determined via Equation (8) [39]:

(8) $$S(\%)=\frac{w_{s}-w_{d}}{w_{d}}\times 100.$$

The equilibrium water content (EWC) of the hydrogel was calculated according to the following equation [33]:

(9) $$EWC(\%)=\frac{w_{EWC}-w_{d}}{w_{d}}\times 100.$$

#### 4.4.4. Kinetics Study

To investigate the water diffusion mechanisms of the cross-linked hydrogels, the first-order swelling constant (*k*_1,*S*_) and the second-order swelling constant (*k*_2,*S*_) were used [40,41]:

(10) $$\frac{dS}{dt}=k_{1,S}\left(S_{max.}-S\right),$$

(11) $$\frac{dS}{dt}=k_{2,S}{\left(S_{max.}-S\right)}^{2}.$$

Equations (10) and (11) are modified as follows by applying the initial conditions, *S* = 0 at *t* = 0, and *S* = *S* at *t* = *t*:

(12) $$\ln W=k_{1,S}t,$$

(13) $$W=\frac{S_{max}}{S_{max}-S},$$

(14) $$\;\frac{t}{S}=A+Bt,$$

(15) $$A=r_{0}=\frac{1}{k_{2,S}\times S_{max.}^{2}},$$

(16) $$B=\frac{1}{S_{max.}},$$

where *S_max._* is the theoretical degree of swelling at equilibrium; *W* is the water absorption capacity; *A* is the reverse of the initial swelling rate; *B* is the reverse of the equilibrium swelling degree; and r_0_ is the initial swelling rate.

The water diffusion nature of the hydrogels can be examined using the following formulas for the first 60% of the swelling curves [42]:

(17) $$F_{swp}=\frac{M_{t}-M_{0}}{M_{0}}=kt^{n},$$

(18) $$\ln F_{swp}=n\ln t+\ln k,$$

where *M_t_* and *M_0_* are the weights of the swollen and dry hydrogel samples at time t (min), k is the specific constant of hydrogel, and n determines the mechanism of water diffusion.

The diffusion coefficient (*D*) is another parameter that is used to evaluate their swelling behavior. The short-time approximation method was used to determine this parameter, and this is only relevant for the initial 60% of the swelling ratio [33].

(19) $$F=4{\left[\frac{D}{\pi r^{2}}\right]}^{1/2}t^{1/2},$$

where *r* (cm) is the radius of the cylindrical hydrogel samples; *t* (s) is time; and *D* (cm^2^/s) is the diffusion coefficient.

#### 4.4.5. Chemical Structure Analysis (FTIR)

The Fourier transform infrared (FTIR) spectra of native polymers and e-beam-synthesized hydrogels were recorded with a Perkin Elmer (Waltham, MA, USA) Spectrum 100 instrument in the 4000–600 cm^–1^ range with 64 scans/sample and 4 cm^–1^ resolution to confirm the hydrogels composition and shifting of bands position. After the swelling analysis, the hydrogels were oven-dried to constant mass and used further for FTIR investigation. The FTIR instrument is equipped with a diamond crystal Miracle ATR accessory (Pike Technologies, Madison WI, USA). Spectra were processed with Spectrum software v. 6.3.2 using normalization and baseline correction.

#### 4.4.6. Hydrogel Microstructure

The microstructure of the hydrogels was observed using the scanning electron microscopy (SEM) FEI Quanta Inspect S model (FEI Company, Hillsboro, OR, USA). Before the SEM investigations, the hydrogel samples were swollen to equilibrium in distilled water and then freeze-dried. Following the gold coating, the cross-section of the freeze-dried hydrogels was examined. The images were acquired at a magnification of ×50,000 and an acceleration voltage of 20 kV.

#### 4.4.7. Statistical Analysis

All experimental data were carried out in triplicate, and all values were presented as the mean value and standard deviation (SD) of three independent samples (n = 3).

## Figures and Tables

**Table 1:** Hydrogel sizes and their physical properties.

Solution Codes	Hydrogel Formulations	Diameter of Wet Hydrogels	Diameter and Height of Dry Hydrogels		Weight (g)	Density (g/cm^3^)
		Ø (mm)	Ø (mm)	h (mm)		
I-20	I_15_-20	15	12.37 ± 0.18	1.41 ± 0.13	0.2377 ± 0.02	1.416 ± 0.023
	I_10_-20	10	7.61 ± 0.55	1.32 ± 0.24	0.0871 ± 0.03	
	I_7_-20	7	5.15 ± 0.22	1.15 ± 0.08	0.0377 ± 0.01	
II-20	II_15_-20	15	12.82 ± 0.35	1.30 ± 0.04	0.2032 ± 0.01	1.225 ± 0.013
	II_10_-20	10	8.89 ± 0.25	1.47 ± 0.12	0.1113 ± 0.01	
	II_7_-20	7	5.51 ± 0.28	1.19 ± 0.15	0.0412 ± 0.01	
I-40	I_15_-40	15	12.60 ± 0.45	3.41 ± 0.14	0.5093 ± 0.01	1.181 ± 0.034
	I_10_-40	10	8.09 ± 0.20	3.57 ± 0.10	0.2102 ± 0.01	
	I_7_-40	7	5.24 ± 0.31	3.91 ± 0.29	0.0804 ± 0.01	
II-40	II_15_-40	15	12.24 ± 0.42	3.41 ± 0.25	0.4983 ± 0.07	1.172 ± 0.092
	II_10_-40	10	8.38 ± 0.24	3.57 ± 0.03	0.2182 ± 0.04	
	II_7_-40	7	5.90 ± 0.33	3.93 ± 0.20	0.1013 ± 0.01	

**Table 2:** The gel fraction (G %), the swollen polymer fraction (ν_2s_), the average molecular weight between crosslinks (M_C_), crosslinking density ($\rho_{x}$), mesh size (ξ), and porosity of hydrogels (P%).

Code Samples	G (%)	ν_2s_ × 10^–3^ (cm^3^)	Mc (kg/mol)	$\rho_{x}\times$10^–3^ (mol/cm^3^)	ξ (nm)	P (%)
I_15_-20	84.10 ± 0.09	9.36 ± 0.53	60.79 ± 5.85	1.22 ± 0.12	77.34 ± 5.19	98.49 ± 0.30
I_10_-20	83.17 ± 4.09	5.52 ± 0.94	152.53 ± 43.35	0.34 ± 0.10	146.36 ± 29.24	99.30 ± 0.17
I_7_-20	83.56 ± 0.32	3.27 ± 0.20	358.20 ± 36.56	0.21 ± 0.02	266.65 ± 19.03	99.51 ± 0.05
I_15_-40	93.73 ± 0.63	21.08 ± 0.03	12.70 ± 0.03	5.83 ± 0.01	26.96 ± 0.04	97.52 ± 0.11
I_10_-40	91.23 ± 0.22	12.87 ± 0.45	29.48 ± 1.75	2.51 ± 0.15	48.42 ± 2.00	98.51 ± 0.10
I_7_-40	90.12 ± 0.17	9.09 ± 0.18	53.07 ± 1.74	1.39 ± 0.05	72.95 ± 1.67	98.95 ± 0.07
II_15_-20	84.79 ± 0.08	10.62 ± 0.06	42.26 ± 0.43	1.78 ± 0.02	61.84 ± 0.43	98.67 ± 0.02
II_10_-20	88.51 ± 0.85	7.80 ± 0.06	71.24 ± 0.93	1.05 ± 0.01	89.00 ± 0.81	99.08 ± 0.07
II_7_-20	86.57 ± 3.94	4.60 ± 0.41	142.90 ± 19.37	0.43 ± 0.06	144.50 ± 13.70	99.40 ± 0.07
II_15_-40	90.55 ± 0.49	16.49 ± 1.57	19.36 ± 3.13	3.93 ± 0.64	36.18 ± 4.08	97.83 ± 0.38
II_10_-40	90.28 ± 0.39	12.91 ± 0.38	29.10 ± 1.46	2.58 ± 0.13	48.10 ± 1.68	98.55 ± 0.15
II_7_-40	90.38 ± 0.26	10.18 ± 0.388	43.53 ± 2.78	1.73 ± 0.11	63.67 ± 2.83	98.84 ± 0.08

**Table 3:** Swelling degree and equilibrium water content of hydrogels.

Code Hydrogels	S_max_. (%)	EWC (%)
I_15_-20	7489 ± 1232	98.52 ± 0.28
I_10_-20	16,472 ± 2822	99.30 ± 0.17
I_7_-20	20,441 ± 2204	99.51 ± 0.05
I_15_-40	4040 ± 188	97.58 ± 0.11
I_10_-40	6740 ± 449	98.53 ± 0.09
I_7_-40	9580 ± 615	98.96 ± 0.06
II_15_-20	7528 ± 132	98.69 ± 0.02
II_10_-20	10,875 ± 856	99.09 ± 0.07
II_7_-20	16,757 ± 2019	99.40 ± 0.07
II_15_-40	4703 ± 791	97.88 ± 0.16
II_10_-40	6949 ± 740	98.57 ± 0.14
II_7_-40	8633 ± 615	98.85 ± 0.08

**Table 4:** The values of first-order swelling rate constants (swelling rate constants, k_1,S_ × 10^3^/min^−1^), second-order swelling rate constants (k_2,S_ × 10^8^/g × gel (g × water × min)^−1^, r_o_/g × water (g × gel × min)^−1^), and equilibrium swelling (Seq/g × water (g × gel)^−1^).

Code	First-Order Rate Constants		Second-Order Rate Constants			
	$k_{1,s}$	$R^{2}$	$k_{2,s}$	$r_{0}$	Seq.	$R^{2}$
I_15_-20	0.491	0.996	6.036	0.200	9093	0.993
I_10_-20	0.371	0.995	1.543	0.145	21,142	0.994
I_7_-20	0.291	0.986	0.747	0.156	29,272	0.977
I_15_-40	0.304	0.992	3.744	0.832	5665	0.938
I_10_-40	0.300	0.993	2.138	0.534	9354	0.901
I_7_-40	0.379	0.978	2.392	0.271	12,410	0.959
II_15_-20	0.517	0.991	5.875	0.222	8756	0.994
II_10_-20	0.395	0.986	2.331	0.224	13,833	0.987
II_7_-20	0.373	0.981	1.338	0.153	22,094	0.978
II_15_-40	0.390	0.990	5.310	0.530	5960	0.972
II_10_-40	0.400	0.986	3.567	0.373	8665	0.944
II_7_-40	0.478	0.969	3.864	0.244	10,296	0.961

**Table 5:** The values of swelling exponents n, swelling constants k, and diffusional coefficient (D × 10^3^/cm^2^s^−1^) in different hydrogel systems, cross-linked by e-beam irradiation to a dose of 12.5 kGy.

Code	Swelling Diffusion			Diffusional Coefficient	
	n	k	*R* ^2^	D	*R* ^2^
I_15_-20	0.788	0.128	0.994	2.346	0.995
I_10_-20	0.834	0.160	0.997	2.787	0.989
I_7_-20	0.745	0.291	0.996	1.095	0.994
I_15_-40	0.695	0.080	0.996	0.206	0.998
I_10_-40	0.686	0.138	0.994	0.199	0.998
I_7_-40	0.611	0.401	0.989	0.223	0.995
II_15_-20	0.680	0.264	0.997	1.474	0.990
II_10_-20	0.743	0.196	0.985	1.134	0.999
II_7_-20	0.704	0.369	0.988	0.931	0.999
II_15_-40	0.659	0.148	0.997	0.343	0.999
II_10_-40	0.607	0.310	0.991	0.272	0.994
II_7_-40	0.538	0.704	0.979	0.225	0.992

**Table 6:** Detailed components of the hydrogel.

Solution Codes	Reagents/100 mL Solution (g)				Dose (kGy)
	NaAlg	AAc	PEO	K_2_S_2_O_8_	
I	0.5	20	0.1	0.1	12.5
II	1	20	0.1	0.1	12.5

**Table 7:** The volume of polymeric mixtures poured into Petri dishes and the thickness of hydrogel films after cross-linking and drying.

Solution Codes	Amounts (mL) (Petri Dish, Ø = 90 mm)	Film Thickness (mm)
I-20	20	1.53 ± 0.05
II-20		1.88 ± 0.10
I-40	40	3.80 ± 0.21
II-40		4.92 ± 0.07

## Data Availability

The original contributions presented in the study are included in the article, further inquiries can be directed to the corresponding authors.

## References

[1] Hoffman A.S. (2001). Hydrogels for Biomedical Applications. Ann. N. Y. Acad. Sci..

[2] Ullah F., Othman M.B.H., Javed F., Ahmad Z., Akil H.M. (2015). Classification, Processing and Application of Hydrogels: A Review. Mater. Sci. Eng. C.

[3] Raza M.A., Jeong J.-O., Park S.H. (2021). State-of-the-Art Irradiation Technology for Polymeric Hydrogel Fabrication and Application in Drug Release System. Front. Mater..

[4] Craciun G., Calina I.C., Demeter M., Scarisoreanu A., Dumitru M., Manaila E. (2023). Poly(Acrylic Acid)-Sodium Alginate Superabsorbent Hydrogels Synthesized by Electron Beam Irradiation Part I: Impact of Initiator Concentration and Irradiation Dose on Structure, Network Parameters and Swelling Properties. Materials.

[5] Stachowiak N., Kowalonek J., Kozlowska J., Burkowska-But A. (2023). Stability Studies, Biodegradation Tests, and Mechanical Properties of Sodium Alginate and Gellan Gum Beads Containing Surfactant. Polymers.

[6] Rosiak J.M., Ulański P. (1999). Synthesis of Hydrogels by Irradiation of Polymers in Aqueous Solution. Radiat. Phys. Chem..

[7] Fazolin G.N., Varca G.H.C., de Freitas L.F., Rokita B., Kadlubowski S., Lugão A.B. (2020). Simultaneous Intramolecular Crosslinking and Sterilization of Papain Nanoparticles by Gamma Radiation. Radiat. Phys. Chem..

[8] Kowalski G., Witczak M., Kuterasiński Ł. (2024). Structure Effects on Swelling Properties of Hydrogels Based on Sodium Alginate and Acrylic Polymers. Molecules.

[9] Dolbow J., Fried E., Ji H. (2004). Chemically Induced Swelling of Hydrogels. J. Mech. Phys. Solids.

[10] Tran V.T., Mredha T.I., Pathak S.K., Yoon H., Cui J., Jeon I. (2019). Conductive Tough Hydrogels with a Staggered Ion-Coordinating Structure for High Self-Recovery Rate. ACS Appl. Mater. Interfaces.

[11] Waham Ashaier Laftah S.H., Ibrahim A.N. (2011). Polymer Hydrogels: A Review. Polym. Plast. Technol. Eng..

[12] Redy Keisar O., Nahum V., Yehezkel L., Marcovitch I., Columbus I., Fridkin G., Chen R. (2021). Active and Strippable PVA/Borax/NaBO3 Hydrogel for Effective Containment and Decontamination of Chemical Warfare Agents. ACS Omega.

[13] Niu B., Jia J., Wang H., Chen S., Cao W., Yan J., Gong X., Lian X., Li W., Fan Y.-Y. (2019). In Vitro and in Vivo Release of Diclofenac Sodium-Loaded Sodium Alginate/Carboxymethyl Chitosan-ZnO Hydrogel Beads. Int. J. Biol. Macromol..

[14] Golafshan N., Rezahasani R., Tarkesh Esfahani M., Kharaziha M., Khorasani S.N. (2017). Nanohybrid Hydrogels of Laponite: PVA-Alginate as a Potential Wound Healing Material. Carbohydr. Polym..

[15] Tao L., Shi C., Zi Y., Zhang H., Wang X., Zhong J. (2024). A Review on the Chemical Modification of Alginates for Food Research: Chemical Nature, Modification Methods, Product Types, and Application. Food Hydrocoll..

[16] Mukherjee K., Dutta P., Saha A., Dey S., Sahu V., Badwaik H., Giri T.K. (2024). Alginate Based Semi-IPN and IPN Hydrogel for Drug Delivery and Regenerative Medicine. J. Drug Deliv. Sci. Technol..

[17] Haryanto, Singh D., Han S.S., Son J.H., Kim S.C. (2015). Poly(Ethylene Glycol) Dicarboxylate/Poly(Ethylene Oxide) Hydrogel Film Co-Crosslinked by Electron Beam Irradiation as an Anti-Adhesion Barrier. Mater. Sci. Eng. C.

[18] Kopka B., Kost B., Rajkowska K., Pawlak A., Kunicka-Styczyńska A., Biela T., Basko M. (2021). A Simple Strategy for Efficient Preparation of Networks Based on Poly(2-Isopropenyl-2-Oxazoline), Poly(Ethylene Oxide), and Selected Biologically Active Compounds: Novel Hydrogels with Antibacterial Properties. Soft Matter.

[19] Aizaz A., Nawaz M.H., Ismat M.S., Zahid L., Zahid S., Ahmed S., Abbas M., Vayalpurayil T., Rehman M.A.U. (2024). Development and Characterization of Polyethylene Oxide and Guar Gum-Based Hydrogel; a Detailed in-Vitro Analysis of Degradation and Drug Release Kinetics. Int. J. Biol. Macromol..

[20] Haryanto, Singh D., Huh P.H., Kim S.C. (2016). Hyperbranched Poly(Glycidol)/Poly(Ethylene Oxide) Crosslinked Hydrogel for Tissue Engineering Scaffold Using e-Beams. J. Biomed. Mater. Res. A.

[21] Peters J.T., Wechsler M.E., Peppas N.A. (2021). Advanced Biomedical Hydrogels: Molecular Architecture and Its Impact on Medical Applications. Regen. Biomater..

[22] Erceg T., Vukić N. (2023). Architecture of Hydrogels. Hydrogels, Fundamentals to Advanced Energy Applications.

[23] Ghobashy M.M., Bassioni G. (2018). PH Stimuli-Responsive Poly(Acrylamide-Co-Sodium Alginate) Hydrogels Prepared by γ-Radiation for Improved Compressive Strength of Concrete. Adv. Polym. Technol..

[24] Bardajee G.R., Hooshyar Z., Zehtabi F., Pourjavadi A. (2012). A Superabsorbent Hydrogel Network Based on Poly((2-Dimethylaminoethyl) Methacrylate) and Sodium Alginate Obtained by γ-Radiation: Synthesis and Characterization. Iran. Polym. J..

[25] Hariharan D., Peppas N.A. (1996). Characterization, Dynamic Swelling Behaviour and Solute Transport in Cationic Networks with Applications to the Development of Swelling-Controlled Release Systems. Polymer.

[26] Zaman K., Dahlan M., Hashim K., Ghazali Z. (2004). The Application of Electron Accelerator: Liquid, Thin Film and Gases.

[27] Hashim K., Yacob N. (2004). Radiation Processing of Sago Hydrogel Thin Film.

[28] Varaprasad K., Nùñez D., Ide W., Jayaramudu T., Sadiku E.R. (2020). Development of High Alginate Comprised Hydrogels for Removal of Pb(II) Ions. J. Mol. Liq..

[29] Işık B. (2004). Swelling Behavior and Determination of Diffusion Characteristics of Acrylamide–Acrylic Acid Hydrogels. J. Appl. Polym. Sci..

[30] Yi X., Xu Z., Liu Y., Guo X., Ou M., Xu X. (2017). Highly Efficient Removal of Uranium(vi) from Wastewater by Polyacrylic Acid Hydrogels. RSC Adv..

[31] Pucić I., Jurkin T. (2012). FTIR Assessment of Poly(Ethylene Oxide) Irradiated in Solid State, Melt and Aqeuous Solution. Radiat. Phys. Chem..

[32] Nagasawa N., Yagi T., Kume T., Yoshii F. (2004). Radiation Crosslinking of Carboxymethyl Starch. Carbohydr. Polym..

[33] Karadag E., Saraydin D., Sahiner N., Güven O. (2001). Radiation Induced Acrylamide/Citric Acid Hydrogels and Their Swelling Behaviors. J. Macromol. Sci. Part A.

[34] Yiamsawas D., Kangwansupamonkon W., Chailapakul O., Kiatkamjornwong S. (2007). Synthesis and Swelling Properties of Poly[Acrylamide-Co-(Crotonic Acid)] Superabsorbents. React. Funct. Polym..

[35] Ding Z.Y., Aklonis J.J., Salovey R. (1991). Model Filled Polymers. VI. Determination of the Crosslink Density of Polymeric Beads by Swelling. J. Polym. Sci. B Polym. Phys..

[36] Gudeman L.F., Peppas N.A. (1995). PH-Sensitive Membranes from Poly(Vinyl Alcohol)/Poly(Acrylic Acid) Interpenetrating Networks. J. Membr. Sci..

[37] Lee B.H., Li B., Guelcher S.A. (2012). Gel Microstructure Regulates Proliferation and Differentiation of MC3T3-E1 Cells Encapsulated in Alginate Beads. Acta Biomater..

[38] Liu D., Yang M., Wang D., Jing X., Lin Y., Feng L., Duan X. (2021). DPD Study on the Interfacial Properties of PEO/PEO-PPO-PEO/PPO Ternary Blends: Effects of Pluronic Structure and Concentration. Polymers.

[39] Chen J., Park H., Park K. (1999). Synthesis of Superporous Hydrogels: Hydrogels with Fast Swelling and Superabsorbent Properties. J. Biomed. Mater. Res..

[40] Üzüm Ö.B., Karadağ E. (2006). A New Sorbent Chemically Cross-Linked Highly Swollen Copolymeric Hydrogels for Dye Uptake. Polym. Plast. Technol. Eng..

[41] Peppas N.A., Franson N.M. (1983). The Swelling Interface Number as a Criterion for Prediction of Diffusional Solute Release Mechanisms in Swellable Polymers. J. Polym. Sci. Polym. Phys. Ed..

[42] Karadaǧ E., Üzüm Ö.B., Saraydin D. (2002). Swelling Equilibria and Dye Adsorption Studies of Chemically Crosslinked Superabsorbent Acrylamide/Maleic Acid Hydrogels. Eur. Polym. J..

